# Healthcare in a pure gatekeeping system: utilization of primary, mental and emergency care in the prison population over time

**DOI:** 10.1186/s40352-021-00136-8

**Published:** 2021-05-13

**Authors:** Jacques Spycher, Mark Dusheiko, Pascale Beaupère, Bruno Gravier, Karine Moschetti

**Affiliations:** 1 Center for Primary Care and Public Health (Unisanté), University of Lausanne, Lausanne, Switzerland; 2 University of Lausanne, Lausanne, Switzerland; 3 Service of Correctional Medicine and Psychiatry (SMPP), University hospital of Lausanne (CHUV), Lausanne, Switzerland; 4 Technology Assessment Unit (UET), University hospital of Lausanne (CHUV), Lausanne, Switzerland

**Keywords:** Health service research, Prison, Multilevel and dynamic modelling, Primary care, Nursing, Mental health and emergency care, Ethnic differences in health care

## Abstract

**Background:**

This study investigates the prisoner and prison-level factors associated with healthcare utilization (HCU) and the dynamic effects of previous HCU and health events. We analyze administrative data collected on annual adult prisoner-stay HCU (*n* = 10,136) including physical and mental chronic disease diagnoses, acute health events, penal circumstances and prison-level factors between 2013 and 2017 in 4 prisons of Canton of Vaud, Switzerland. Utilization of four types of health services: primary, nursing, mental and emergency care; are assessed using multivariate and multi-level negative binomial regressions with fixed/random effects and dynamic models conditional on prior HCU and lagged health events.

**Results:**

In a prison setting with health screening on detention, removal of financial barriers to care and a nurse-led gatekeeping system, we find that health status, socio-demographic characteristics, penal history, and the prison environment are associated with HCU overtime. After controlling for chronic and past acute illnesses, female prisoners have higher HCU, younger adults more emergencies, and prisoners from Africa, Eastern Europe, and the Americas lower HCU. New prisoners, pretrial detainees or repeat offenders utilize more all types of care. Overcrowding increases primary care but reduces utilization of mental and emergency services. Higher expenditure on medical staff resources is associated with more primary care visits and less emergency visits. The dynamics of HCU across types of care shows persistence over time related to emergency use, previous somatic acute illnesses, and acting out events. There is also evidence of substitution between psychiatric and primary care.

**Conclusions:**

The prison healthcare system provides an opportunity to diagnose and treat unmet health needs for a marginalized population. Access to psychiatric and chronic disease management during incarceration and prevention of emergency or acute events can reduce future demand for care. Prioritization of high-risk patients and continuity of care inside and outside of prisons may reduce public health pressures in the criminal system. The prison environment and prisoners’ penal circumstances impacts healthcare utilization, suggesting better coordination between the criminal justice and prison health systems is required.

**Supplementary Information:**

The online version contains supplementary material available at 10.1186/s40352-021-00136-8.

## Background

Prisons face a significant challenge to meet the healthcare needs of prisoners who have a high prevalence of infectious, chronic physical and mental illnesses, substance abuse problems as well as multi-morbidities (Abbott, Magin, & Hu, 2016; Binswanger, Krueger, & Steiner, 2009; Fazel & Baillargeon, 2011; Fazel & Seewald, 2012; Moschetti et al., 2015; Wilper et al., 2009; Wolff et al., 2011). Significant unmet needs prevail due to low socioeconomic status, precarious life experience, and limited access to healthcare prior to incarceration (Green, Foran, & Kouyoumdjian, 2016; Wilper et al., 2009).

The number of prisoners has been increasing in more than two-thirds of countries worldwide with more than 10.74 million people held in penal institutions in 2015 (Walmsley, 2018). In the USA, the prison population grew by 12% between 2000 and 2015 (Walmsley, 2018). In Switzerland, the number of incarcerated individuals increased by 20% between 2000 and 2017 (Swiss Federal Statistics Office, 2018). This growth is leading to prison over-population (Dünkel, 2017; Sturge, 2018) that may weaken sanitary conditions, increase spread of diseases and stress amongst inmates (MacDonald, 2018). Longer sentences imply a rise in the average age of prisoners and associated diseases that adds to the complexity of their health needs (Williams, Stern, Mellow, Safer, & Greifinger, 2012).

Penitentiary health systems differ in whether responsibility for healthcare lies within the prison service and justice system, or with the health authority. They face organizational and resource constraints affecting access to onsite care and use of outside care facilities. It can be difficult for prison services to compete for staff and resources with established healthcare providers, hence mandating responsibility to local public healthcare providers can ensure independence of healthcare from the prison service, better accessibility as well as integration of care (see chapter 4 in (Mistiaen et al., 2017)). Funding and provision of prison health services tends to be in the public sector. However, budget cuts resulting in reductions in staff and competitive pressure to contain costs, has seen an increase in privatization of prison care in some countries (Ismail, 2020). This has raised concerns about excessive cost reductions at the expense of quality of care, a lack of transparency and problems of oversite (Moore, 1998). Since November 2018, there is a tendency for some Swiss prison administrations to ask prisoners to finance part of their health care (Conférence latine des Chefs des Départements de justice et police, 2008), which may raise concerns about assuring equality of access and equivalence of care within prisons (Mansour, 2019).

The increase in the prison population with rising healthcare needs, pressures from over-crowding and resource limits on healthcare services, implies a necessity to monitor utilization of care services over-time and understand the prisoner characteristics associated with utilization of the different care services to identify those inmates in higher needs. The influence of the prison environment and level of healthcare resources on utilization patterns is of interest as this can facilitate the planning and organization of services and increase transparency to ensure quality, efficiency and equity of care services for a marginalized population.

Most of the literature on healthcare utilization (HCU) and its determinants in prison relies on cross-sectional designs and shows greater use than in the general population (Feron, Paulus, Tonglet, Lorant, & Pestiaux, 2005; Marshall, Simpson, & Stevens, 2001; Twaddle, 1976), with high rates of primary care utilization (Moschetti et al., 2017), but also of emergency care treatments (Tuinema, Orkin, Cheng, Fung, & Kouyoumdjian, 2019)*.* Physical symptoms, mental health disorders and substance abuse as well as treatment prior to incarceration and demographic characteristics are good predictors of ambulatory care (Gonçalves, Gonçalves, Martins, & Dirkzwager, 2014; Nobile, Flotta, Nicotera, Pileggi, & Angelillo, 2011; Nowotny, 2016; Wangmo et al., 2016). In the Swiss context, there is also evidence that prison facility-level occupancy rate is associated with primary care and ambulatory psychiatric HCU (Moschetti et al., 2017). There is paucity of data on emergency care utilization in the prison system and little is reported on changes in HCU during imprisonment. However, for a large part of the inmate population, incarceration lasts several years, and across multiple spells in prison. HCU is likely to be dynamic as health status and healthcare utilization may change over time especially with their duration in prison, their previous use of services and adverse health events. One study investigating young prisoners in Portugal finds that physical HCU decreased with time spent in prison (Goncalves et al., 2017). Overall, longitudinal studies on HCU are scarce for this population.

Outside of prison, marginalized populations, such as the uninsured, utilize emergency departments more than routine care (Zhou, Baicker, Taubman, & Finkelstein, 2017). Even in health care systems with universal coverage and comprehensive access to care, inequities in HCU exist, particularly for the uptake of preventive care and specialist consultations (Cookson, Propper, Asaria, & Raine, 2016). HCU is characterized by both over-use of low value care and under-use of effective care (Chandra, Handel, & Schwartzstein, 2019). Unmet health needs have been associated predominantly with patient characteristics (Aragon, Chalkley, & Goddard, 2017). Education and psychological traits have been associated with sub-optimal utilization, responsiveness to medical information, uptake of preventive care and compliance with treatment (Borboudaki et al., 2020; Cutler & Lleras-Muney, 2008). A minority of medically and socially complex patients account for a high proportion of HCU, with significant persistence in utilization over time (Ellis, Martins, & Rose, 2018). Improvements in the accessibility and quality of primary care can reduce avoidable emergency department visits, although interventions targeting patients frequently admitted to hospital have had mixed results in reducing hospitalizations (Finkelstein, Zhou, Taubman, & Doyle, 2020; Harrison et al., 2014). The organization of the health system and social environment significantly influence access to care, and choice of providers. The treating medical practitioner influences utilization and differences in medical beliefs about treatment effectiveness, physician practice styles (e.g. interventionist), financial incentives and structures such as managed or integrated care are important determinants of HCU (Cutler, Skinner, Stern, & Wennberg, 2019; Finkelstein, Gentzkow, & Williams, 2016).

This study investigates the associations between healthcare utilization and prisoner health, demographic and social factors, penal circumstances as well as the prison environment. We adapt Andersen’s conceptual framework for HCU determinants to the prison environment of the canton of Vaud in Switzerland to examine utilization of four types of services, namely, nurse and general practitioners (GP) consultations, mental and emergency HCU. Unlike in the community setting, financial barriers to access such as the cost of health care are removed, timely access to primary care and specialist psychiatric care is assured, and a comprehensive health screening upon incarceration identifies the presence of chronic illness and potential unmet health needs. Hence, we are able to investigate the specific role of the multiple need factors, demographic, ethnic and socio-economic backgrounds for this vulnerable population, independently of otherwise important factors affecting HCU in the community. The prison healthcare system is organized as a nurse-led gatekeeping system with continuity of care between primary and mental health care professionals. By analyzing longitudinal data over a period of 5 years (2013–2017) across four prisons, we investigate the dynamics of care utilization across these four types of services in relation to time spent in the prison and previous health events.

### Setting

Governed by federal laws, the Swiss prison system is organized and managed at the cantonal (state) level, implying that the prison systems differ substantially across cantons. However, the principle of equivalence of care established by international norms (Elger, 2008; Jotterand & Wangmo, 2014; UNODC, 2020; WHO, 2019), and governed by the national medical and ethical guidelines, applies in all Swiss prisons (Swiss Academy of Medical Sciences, 2015).

The prison system in canton of Vaud is managed by the public administration. There are four prisons for adults, and a fifth prison that houses minors and young adults (not included in this study). Bois-Mermet and Croisée prisons house mainly pretrial (remand) prisoners, and Tuilière houses some male remand prisoners and all female prisoners in separate sections. The Plaine de l’Orbe prison houses prisoners with long sentences.

Healthcare service provision in prison is independent of the judiciary and penitentiary authorities since 1995 (UNODC, 2020) and is managed by the Service of Correctional Medicine and Psychiatry (SMPP), belonging to the psychiatric department of the university hospital of the canton of Vaud. Prison staff and healthcare professionals are salaried employees and services are public (exception for some emergency services). Each prison has an on-site outpatient clinic where nurses, GPs, psychiatrists, and some specialists like dermatologists, dentists as well as psychologists are available.

On-site healthcare services in prisons are reimbursed by health insurance, which is mandatory for Swiss residents. For individuals with health insurance before incarceration, the insurance is financed using private funds whenever possible. For prisoners without the means to pay (illegal residents or prisoners in a precarious socioeconomic situation), the prison administration either bears the medical costs directly, or obtains subsidies from the canton’s social insurance fund to purchase health insurance. In the context of this analysis, there are virtually no financial barriers of access to care for patients in prison.

**Fig. 1:**
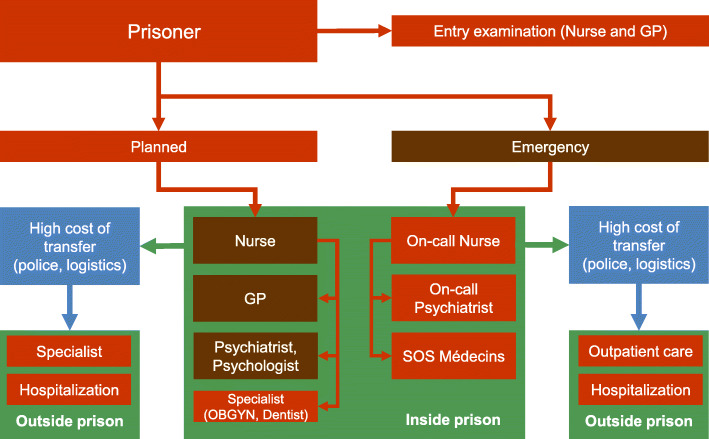
Prisoner management and access to healthcare services in the canton of Vaud. SMPP: Service of Correctional Medicine and Psychiatry; GP: general practitioner, OBGYN: obstetrician and gynecologists, SOS Médecins: on-call GPs for on- site care

Figure 1 summarizes an inmate’s healthcare pathway, distinguishing planned and emergency care. Access to health services is close to that of a managed care organization, where the nurse plays a key gatekeeping role managing on-site access to primary, mental (psychiatrists and psychologists) and specialist care. In close collaboration with doctors, nurses organize consultations, provide daily follow-up care, and coordinate transfer for treatment outside the facilities and then coordination with police and hospital security. In the case of emergency out of hours care, the on-call nurse must assess the need to contact the on-call GP or psychiatrist for on-site care, or to transfer the inmate to the cantonal hospital emergency room.

## Methods

### Data description

The study uses anonymized longitudinal administrative data between 2013 and 2017 of all adult inmates already incarcerated on January 1, 2013 and those who entered or re-entered at any time between 2013 and 2017 in the four closed prisons of Canton of Vaud. The bespoke database collected by the SMPP routinely extracts selected data from the prisoner and patient record for monitoring trends in prisoner characteristics, health status and healthcare utilization in the prisons. Data contain detailed information on HCU, demographics, social situation, health status, penal circumstances and duration of prisoner stays. The unit of observation is every prison stay during the same calendar year, which we define as the year-stay. An individual may have one or several year-stays during the same calendar year or year-stays that cover several calendar years. For example, a prison sentence executed from 1st July 2014 to 31st July 2015 generates 2 year-stays (1st July to 31st December 2014 and 1st January to 31st July 2015). After cleaning the data and keeping stays for inmates with a medical examination upon entry (82% of initial observations), the panel includes 10,136 year-stays, effectuated by 4928 prisoners, over the 5 years. For the dynamic analysis the sample is restricted to prisoners with repeated observations, that is with at least 2 year-stays over the 5 years (sample size = 5208).

### Conceptual framework

Anderson’s framework and its extensions (Aday & Andersen, 1974; Andersen, 1995; Andersen & Newman, 1973; Andersen & Newman, 2005), model the factors determining HCU in a community setting according to four categories: predisposing (demographic variables, socioeconomic status), enabling (insurance coverage, income, education), need (illness level) and environmental (organization of the healthcare system, social, geographic and climatic factors). Additional categories playing a role in the HCU have been identified, including cultural and vulnerability factors (Gelberg, Andersen, & Leake, 2000; Janssen, Swart, & von Lengerke, 2014)). In the context of the prison setting, the needs factors (perceived and evaluated) and predisposing factors seem to be reasonable dimensions that drive the individual’s propensity to seek healthcare utilization. However, the enabling factors and the health system environment may be less important in the institutionalized setting of prisons in canton Vaud as publicly provided health services ensure uniformity of access. Moreover, screening upon prison entry allows the identification of unmet health problems. Unlike the community setting, distinct penal circumstances and the prison environment need to be considered as these structural pressures can accelerate health deterioration due to deprivation of liberty, social and psychological distress as well as the poor health and safety of daily living conditions.

The prison setting may affect individual values concerning health and the use of health services compared to the community setting. Prisoners valuation and motivation towards improving their health may decline as there are few benefits from improved productivity as earnings from work are limited, and fewer possibilities to enjoy leisure time or consume other goods curtailed, which from a health economic perspective would reduce HCU (Grossman, 2017). The relative costs of health care, however, are low as there are no financial barriers to accessing healthcare services for prisoners in canton Vaud with little opportunity cost of time or alternative means to improve health, which would increase the demand for health care. During incarceration, an individuals’ health, preferences and environment may change with awareness about health problems, medical consultations, education programs or changes in penal as well as prison circumstances, implying the need to consider the dynamics of utilization beyond needs and purely predisposing factors as stated by Andersen’s framework. The extent to which past health shocks and use of health care influences HCU is of interest to understand the persistence of health care needs and to investigate if there is substitution between care services or if it is possible to prevent emergency utilization.

We adapt the behavioral model of HCU developed by Andersen and integrate perspectives from health economic models (Gravelle, Morris, & Sutton, 2012) and the literature analyzing socio-economic inequalities in health care utilization. We then consider four overarching groups of correlates of HCU: the evaluated need factors (prisoners’ health status), predisposing factors (demographic and origin variables), the individual penal circumstances of the prison stays (length of stay, type of crimes, etc. …) and the prison-level factors capturing prison environment.

### Outcome variables: the four measures of healthcare utilization

Healthcare utilization is measured as the total number of routine GP, nurse, mental care visits, and emergency consultations per year-stay. GP, nurse, and mental visits are all ambulatory consultations and provided onsite. The mental care consultations include both visits with psychiatrists, and/or psychologists who closely work with psychiatrists. Emergency care services include, without distinctions, care delivered on site by emergency medical staff and care requiring prisoner transfer to the cantonal hospital.

### Explanatory variables

The prisoner chronic health conditions are indicated by dummy variables for the presence of physical (also referred to as somatic) or/and mental (also referred to as psychiatric) chronic conditions as well as substance abuse problems grouped into categories according to the International Classification of Diseases, version 10 (ICD-10). A detailed description is available in previous research (Moschetti et al., 2015). In addition, a variable counting the number of comorbidities across the specific ICD codes is added in the models.

The predisposing factors encompass gender, age groups and their interactions, marital status and region of origin of prisoners. Moreover, a binary indicator for having health insurance prior to incarceration acts as a proxy for socioeconomic status and prior access to healthcare.

The prisoners’ penal circumstances are individual characteristics related to the stays and include the type of offense the prisoner is serving: violent, sexual, drug related, or other offenses (e.g. arson, theft, traffic infractions); the detention regime (pretrial detainees or convicted). Individuals whose crime is related to severe mental disorders and high risks of recidivism are mandated psychiatric treatment under the Swiss Criminal Code (Swiss Criminal Code, 1937). The number of past prison-stays and total time spent in prison from the first incarceration.

The prison-level factors include three objective measures accounting for heterogeneity in the prison facility conditions and in the structural characteristics of their care units: i) the prison occupancy rate, defined as operating capacity (prisoner population at the start of a given calendar year) divided by a prison’s designated capacity; ii) the yearly in-flow of new prisoners to each facility to account for the turnover of inmates during the year; iii) yearly costs in millions CHF (1 CHF ≈ 1 USD in 2020) of the full-time equivalent workforce in each facility, which measures the available resources allocated for onsite healthcare in each prison.

### Statistical analysis

The analysis relies on the estimations of three types of models of HCU where HCU is measured by the number of visits per year-stays for the 4 types of services and the objective is to identify to what extent prisoner health conditions, predisposing, prisoners’ penal circumstances and prison-level are correlates of using health services. Models M1 (main models) are estimated using all observed prisoner year-stays (*N* = 10,136) and include the four groups of explanatory variables described above, year and prison fixed effects. The latter accounts for differences in unmeasured facility level factors that affect HCU constantly over time. Negative binomial or Poisson regression models are used to model HCU. The distributions account for the count nature of the data (non-negative integers) as well as the skewness and heavy tailed distribution of the outcome variables. The negative binomial model does not impose equidispersion (i.e. the equality of mean and variance as imposed by the Poisson model), which is measured by the significance of the over-dispersion parameter (Wooldrige, 2010). The length of year-stays is included as an exposure variable to adjust for time (in days) opportunity to use health services in prison. In addition, prisoner-level random effects account for unobserved heterogeneity in utilization that persists over time.

As an extension, we run a two-level model that takes into account unobserved variation at prisoner and prison levels where prisoners are nested (models M2). These models are estimated on the full sample using a mixed effects generalized linear model (GLM) regressions with prison and prisoner level random effects and exploit variations between and within prisons over time. Hence, our three prison-level variables associate differences in the environmental circumstances across the 4 different prisons, and over the 5 years with HCU, conditional on unmeasured differences between prisons estimated by the random effect. Negative binomial distributions fit the best for GP, mental and emergency HCU, while the Poisson distribution is chosen for the nursing HCU.

The third group of models (M3) uses past prisoner year-stay information to account for the dynamics of HCU (*N* = 5208). The estimation sample, therefore, only includes prisoners with at least two prisoner-year stays because the models include all M1 variables plus one period lagged variables of both HCU defined as the number of consultations in the previous year-stay for the 4 types of healthcare services, and the number of acute events experienced by inmates during their previous year-stay. Acute events are for somatic diseases (e.g. influenza, bronchitis, trauma lesions, dermatological, digestive and genital problems), for crisis events (e.g. physical aggressions, drug overdose, hunger strikes), and for psychiatrically diagnosed acting out events (suicide attempt, aggression, self-harm). The dynamic models also control for baseline HCU and for length of lagged stay. Acute health events provide important additional information on the health status and risk profile of prisoners, and dynamic specification ensures we avoid the fact that contemporaneous HCU and acute events are simultaneously determined. The statistical analyses were performed using Stata 15.1.

## Results

### Descriptive statistics

Table 1 provides descriptive statistics for the explanatory variables and the 4 measures of HCU for the full year-stays sample. On average, there are 2.4 doctor visits, and 3.5 mental care consultations per year-stay in prison. Nursing care is most frequent with 12.4 visits per stay on average. Almost 1 in 5 annual stays (0.18) required emergency care.

**Table 1:** Descriptive statistics of HCU outcomes and explanatory variables for the pooled sample of year stays

**Healthcare utilization (HCU)**						
**Number of visits**	**Mean**	**p25**	**p50**	**p75**	**p90**	**p95**
Nursing care	12.43	3	8	16	29	41
Mental care	3.509	0	0	4	10	17
GP care	2.379	0	1	3	6	9
Emergency care	0.179	0	0	0	1	1
**Individual characteristics**						
***Pre-disposing***						
**Socio-demographics**	**N/Mean**	**Percent/SD**		**Origin**	**N/Mean**	**Percent/SD**
Health insurance (^b^)	2382	24%		Africa (^c^)	2787	28%
Married	1987	20%		Eastern Europe	2311	23%
Female	628	6%		Switzerland	1693	17%
**Age**				North Africa (^c^)	1510	15%
18 to 24 (ref)	2068	20%		Western Europe (ref)	1226	12%
25 to 39	5676	56%		Middle East	264	3%
40 to 49	1541	15%		America	233	2%
50 and older	851	8%		Asia	111	1%
***Need factors - Health conditions***						
**Chronic addictions**				**Chronic somatic conditions**		
Drugs	2341	23%		Musculoskeletal	1145	11%
Alcohol	862	9%		Skin	1064	11%
Pharmaceuticals	375	4%		Infectious	760	8%
**Chronic mental conditions**				Circulatory	679	7%
Personality	1875	19%		Digestive	659	7%
Neurotic	1551	15%		Endocrine	659	7%
Schizophrenia	507	5%		Respiratory	608	6%
Retardation	233	2%		Nervous	182	2%
Mood	203	2%		**Acute health events (**^**a**^**)**		
Behavioral	142	1%		Somatic acute	0.405	0.652
				Acting out	0.18	0.649
Chronic comorbidities	1.502	1.607		Crisis	0.337	1.139
***Prisoner penal circumstances***						
Convicted	7906	78%		Time in prison in years	1.606	1.863
Pretrial detention	2230	22%		Length of stay in days	148.2	113.7
Mandatory psych. Treat.	345	3%		Number of stays	1.451	0.836
**Stayed in**				**Type of offense**		
Croisée	4298	42%		Drug related	5159	51%
Plaine de l’Orbe	2564	25%		Other offenses (ref)	3629	36%
Bois-Mermet	2108	21%		Sexual	760	8%
Tuilière	1176	12%		Violent	588	6%
**Prison-level characteristics**						
**Croisée (ref)**	**Mean**	**SD**		**Tuilière**	**Mean**	**SD**
Capacity	211			Capacity	81	
Occupancy	1.454	0.156		Occupancy	1.084	0.11
Prison entries in the year	765	125.3		Prison entries in the year	212.6	31.07
Full-time equivalent cost	1.638	0.036		Full-time equivalent cost	1.382	0.063
**Bois-Mermet**				**Plaine de l’Orbe**		
Capacity	100			Capacity	330	
Occupancy	1.7	0		Occupancy	0.872	0.111
Prison entries in the year	335.4	27.03		Prison entries in the year	268.2	41.23
Full-time equivalent cost	1.143	0.06		Full-time equivalent cost	2.432	0.162
**Data characteristics**						
		**N**				**N**
Total observations (stays)		10,136		Total individuals		4,928

N stands for number. For continuous variables, the table reports the mean (standard deviation -SD-)  . (^a^) These variables are only used in the models which include lags, (^b^) prior to incarceration, (^c^) North Africa includes countries on the Mediterranean rim and Africa does not.

The proportion of female stays is 6% and more than half of the prison stays are experienced by individuals younger than 39 years old. Around 15% of stays are for prisoners of North African origin (i.e. countries on the Mediterranean rim), 27% of African origin (i.e. countries not on the Mediterranean rim), and 17% have Swiss origin. Prisoners having health insurance coverage prior to incarceration account for 24% of stays, and married individuals for 20%. In 23% of the stays, drugs abuse is reported with 19% diagnosed with personality disorder. Prisoners are diagnosed with infectious diseases and skin conditions in 8% and 11% of stays respectively. 22% of stays are executed in pretrial detention. Half of the stays are for drug related offenses and on average, total time spent in prison is about 19 months, and the mean length of year-stay is 148 days. The occupancy rate during prison year-stays ranged from 87% to 170% (see Table 1), and the yearly prisoners’ entry flow ranged from 212 to 765 individuals across prison facilities and years. The total salary charges of FTE personnel for the 4 prison facilities amount CHF 5.60 million over the study period.

## Results of regression models

Table 2 presents the results of the four M1 regression models for GP, nursing, mental and emergency HCU, according to the groups of explanatory variables. Results of the multilevel GLM regressions models (M2) are shown in Table 3 where the results for the prison level characteristics are presented (see Additional file 1: Appendix A1 for entire regressions results). Finally, Table 4 shows results of the dynamic regression models (M3) for the acute events and HCU lagged variables (see Additional file 1: Appendix A2 for the entire estimated regression models). In general, the three estimated models exhibit reasonable goodness of fit for individual HCU data with pseudo R^2^s ranging from 0.27 to 0.41 for primary and mental HCU in models M1. Values are lower for emergency care regressions with R^2^s of 0.1 in model M1 and 0.21 for the dynamic model (M3), reflecting the lower incidence and greater randomness of emergency events.

**Table 2:** Results of the M1 regression models of prisoners’ generalist (GP), nursing, mental and emergency healthcare utilization

	GP		Nursing		Mental		Emergency	
	IRR	SE	IRR	SE	IRR	SE	IRR	SE
**Demographics**								
Female	1.209	0.158	1.463***	0.130	2.009***	0.249	2.287***	0.592
Married	1.026	0.033	0.983	0.023	0.969	0.041	1.112	0.109
Health insurance	1.012	0.031	0.939***	0.020	1.127***	0.038	0.939	0.082
**Age**								
25 to 39	1.070**	0.036	1.007	0.024	1.047	0.044	0.849*	0.082
40 to 49	1.090*	0.049	0.962	0.032	0.984	0.055	0.714**	0.099
50 and older	1.112*	0.064	0.982	0.042	0.994	0.069	0.889	0.153
Female 25 to 39	1.034	0.144	0.903	0.087	0.756**	0.100	0.582*	0.165
Female 40 to 49	1.187	0.186	1.041	0.117	0.646***	0.101	0.662	0.223
Female 50 and older	1.365*	0.235	1.051	0.134	0.636**	0.116	0.555	0.220
**Origin (**^**a**^**)**								
Switzerland	0.990	0.047	1.089**	0.038	1.337***	0.068	0.903	0.116
Africa	0.960	0.044	0.859***	0.030	0.495***	0.029	0.505***	0.071
North Africa	1.130**	0.056	1.053	0.039	1.133**	0.063	1.195	0.156
Middle East	1.128	0.100	1.076	0.070	1.073	0.102	1.072	0.246
Eastern Europe	1.009	0.045	0.863***	0.029	0.661***	0.036	0.770**	0.097
America	1.014	0.085	0.951	0.060	0.821**	0.082	0.578**	0.152
Asia	0.829	0.109	0.991	0.097	1.052	0.151	0.708	0.272
**Chronic somatic conditions**								
Infectious	1.590***	0.069	1.153***	0.038	1.191***	0.064	1.333**	0.174
Skin	1.345***	0.044	1.045*	0.026	0.909**	0.038	1.024	0.110
Musculoskeletal	1.382***	0.044	1.066***	0.026	1.039	0.040	1.063	0.107
Digestive	1.371***	0.051	1.149***	0.032	1.060	0.049	1.332**	0.156
Circulatory	1.392***	0.061	1.442***	0.047	0.908*	0.053	2.150***	0.289
Endocrine	1.305***	0.056	1.251***	0.041	1.130**	0.061	1.317*	0.189
Respiratory	1.277***	0.053	1.047	0.033	1.002	0.050	1.077	0.139
Nervous	1.313***	0.084	1.167***	0.055	1.011	0.077	1.239	0.222
**Chronic addictions**								
Alcohol	0.983	0.039	1.022	0.029	1.515***	0.062	0.804*	0.099
Drugs	1.098***	0.037	1.117***	0.027	2.289***	0.086	1.376***	0.131
Pharmaceuticals	0.955	0.052	0.916**	0.035	1.087	0.058	0.899	0.137
**Chronic mental conditions**								
Schizophrenia	0.962	0.055	1.270***	0.050	3.376***	0.178	1.632***	0.256
Mood	1.234***	0.079	1.145***	0.054	2.142***	0.139	1.505**	0.258
Neurotic	1.119***	0.037	1.099***	0.026	2.276***	0.085	1.419***	0.131
Behavioral	1.072	0.087	0.986	0.055	1.761***	0.151	0.478**	0.169
Personality	1.077**	0.036	1.105***	0.027	2.077***	0.076	2.049***	0.190
Retardation	0.936	0.062	1.046	0.050	1.092	0.071	1.229	0.193
*Chronic comorbidities*	1.036**	0.017	1.023*	0.012	0.989	0.019	0.992	0.044
**Year dummies**								
2014	0.850***	0.034	0.947**	0.025	0.945	0.042	0.684***	0.080
2015	0.838***	0.033	0.832***	0.022	1.116**	0.052	0.560***	0.062
2016	0.943	0.037	0.749***	0.021	1.149***	0.052	0.370***	0.043
2017	1.000	0.047	0.671***	0.023	1.166***	0.066	0.474***	0.068
**Prisoner penal circumstances**								
Pretrial detention	1.051*	0.030	1.189***	0.022	1.322***	0.043	1.130	0.092
Mandatory psych. Treat.	0.904	0.056	0.993	0.043	1.192***	0.065	1.009	0.167
Time in prison	0.936***	0.008	0.867***	0.007	0.921***	0.009	0.891***	0.023
Number of stays	1.038**	0.015	1.059***	0.011	1.018	0.017	1.254***	0.045
**Type of offense (**^**b**^**)**								
Violent	0.932	0.051	0.975	0.040	1.097	0.064	0.982	0.149
Sexual	1.045	0.050	1.027	0.037	1.390***	0.070	1.082	0.143
Drug related	0.964	0.026	0.964*	0.018	0.904***	0.028	0.867*	0.065
**Prison dummies (**^**c**^**)**								
Bois-Mermet	1.425***	0.154	1.137*	0.083	1.523***	0.194	3.500***	1.223
Tuilière	1.056	0.150	0.978	0.096	0.966	0.153	3.551***	1.557
Plaine de l’Orbe	1.024	0.251	1.199	0.198	0.384***	0.103	0.569	0.437
**Prison level characteristics**								
Occupancy	1.124	0.167	1.110	0.110	0.330***	0.052	0.715	0.289
Prison entries in the year	1.000	0.000	1.000**	0.000	1.000	0.000	1.001*	0.001
Full-time equivalent cost	1.223	0.188	0.957	0.099	1.547**	0.273	1.836	0.930
Constant	0.010***	0.003	0.027***	0.005	0.009***	0.003	0.001***	0.001
**Observations**	10,136		10,136		10,136		10,136	
Number of individuals	4928		4928		4928		4928	
Pseudo R2	0.339		0.277		0.327		0.095	

Incidence rate ratios (IRR) and standard errors (SE) are reported,*** *p* < 0.01, ** *p* < 0.05, * *p* < 0.1

Reference categories are (^a^) Western Europe, (^**b**^) other offenses, (^**c**^) Croisée

**Table 3:** Results of the multilevel GLM regressions models (M2) for the prison level characteristics

	GP		Nurse		Mental		Emergency	
	IRR	SE	IRR	SE	IRR	SE	IRR	SE
Occupancy	1.334**	0.157	1.282***	0.080	0.416***	0.047	0.558**	0.136
Prison entries in the year	1.000	0.000	1.001***	0.000	1.000**	0.000	1.000	0.000
Full-time equivalent cost	1.128	0.111	1.092	0.061	0.670***	0.048	0.374***	0.061
Observations	10,136		10,136		10,136		10,136	
Distribution	Negbin		Poisson		Negbin		Negbin	
Pseudo R2	0.316		0.235		0.107		0.081	

Incidence rate ratios (IRR) and standard errors (SE) are reported, *** *p* < 0.01, ** *p* < 0.05, * *p* < 0.1.

**Table 4:** Results of the dynamic regression models (M3) for the acute events and HCU lagged variables

	GP		Nurse		Mental		Emergency	
	IRR	SE	IRR	SE	IRR	SE	IRR	SE
Crisis events	0.985*	0.009	0.978***	0.006	0.990	0.009	0.941***	0.020
Acting out events	1.002	0.020	1.036***	0.013	1.051***	0.018	1.170***	0.043
Acute somatic conditions	1.077***	0.022	1.041***	0.015	0.979	0.023	0.976	0.063
GP consultations	1.014***	0.004	1.016***	0.003	1.012**	0.005	1.017	0.012
Nurse consultations	1.002***	0.001	1.000	0.001	0.999	0.001	1.003	0.003
Mental consultations	0.995**	0.002	0.996**	0.002	1.020***	0.002	1.004	0.006
Emergency consultations	1.035**	0.016	1.019*	0.010	0.984	0.016	1.054*	0.032
Length of stay	1.000**	0.000	0.999***	0.000	0.999***	0.000	0.999**	0.001
Baseline visits	1.008***	0.001	1.011***	0.001	1.006***	0.001	1.012***	0.002
Observations	5208		5208		5208		5208	
Number of individuals	2594		2594		2594		2594	
Pesudo R2	0.364		0.411		0.401		0.216	

Incidence rate ratios (IRR) and standard errors (SE) are reported, *** *p* < 0.01, ** *p* < 0.05, * *p* < 0.1.

### Prisoner socio-demographic characteristics

Female inmates display significantly higher rates of nursing, mental and emergency HCU. The frequency of GP visits increases with age, but emergency admissions decline relative to the youngest age-group (18–25). Mental HCU is higher for younger women. Compared to Western European origin, being of Swiss origin is associated with a larger volume of nurse and mental care consultations. Detainees of African, eastern European and American origins have significantly lower nursing, mental and emergency HCU, while inmates from North Africa have higher utilization of GP and mental care. Health insurance prior to incarceration is significantly associated with lower rate of nursing care but more mental care.

### Prisoner chronic health conditions

Chronic somatic conditions are associated with higher volumes of GP and nurse’ consultations. Circulatory diseases double the risk of emergency consultations while infectious, endocrine and digestive diseases significantly increase the risk by around 30%. Only infectious and endocrine diseases are associated with an increase in the rate of mental healthcare.

A number of psychiatric conditions and substance abuse disorders are significantly and positively associated with primary care and emergency care. Drug addiction in particularly is associated with higher volumes of primary and emergency healthcare. Personality disorders double emergency admission risk, while schizophrenia, mood, and neurotic disorders increase it by 50%. An increase in the number of comorbidities is associated with significantly higher rates of GP and nurse HCU.

### Prisoner penal circumstances

Prisoners in pretrial detention are associated with significantly higher volumes of GP, nursing and mental HCU but significantly higher emergency HCU. Total time spent in prison is significantly and negatively associated with HCU after adjusting for the current length of year-stay as an exposure variable. Each additional stay in prison is associated with an increase in the rate of utilization for GP, nursing and emergency healthcare of between 4% to 25%. Prisoners convicted of sexual offences have higher rates of mental healthcare while drug related offenders consume nursing, mental and emergency HCU less frequently relative to inmates convicted for reasons other than violence, sexual crime, or drugs.

### Prison-level characteristics

Results from M1 models (that include prison fixed effects) associate higher occupancy rates with significantly reduced mental care utilization while an increase in new prison entrants is associated with higher nurse and emergency consultations. An increase in expenditure on staff resources significantly increase rates of consultations for mental healthcare.

Multilevel regression models (M2) with prison random effects indicate that higher occupancy levels are associated with significantly more GP and nurse visits, but significantly lower emergency and mental care. Nurse and mental health visits are positively associated with an increase in the number of new prisoners, while higher expenditure on staff was associated with decreases in the rates of mental care and emergency care utilization.

### Dynamic effects of prior acute health events and prior HCU

Previous acute somatic illnesses are significantly associated with greater numbers of GP and nurse consultations in the following stay. Acting out events significantly increase future nursing, mental and emergency HCU. The use of one healthcare service in the previous stay shows significant persistence in utilization of GP, mental, and emergency healthcare. There are cross sector spillovers with past GP HCU significantly associated with higher nursing and mental care consultations. Past nursing and emergency HCU significantly increase GP visits the following year. Conversely, past mental HCU is associated with significant reductions in primary care consultations.

## Discussion

This study exploits a comprehensive longitudinal database for monitoring healthcare delivery in the canton of Vaud, Switzerland. It investigates prisoner and prison-level determinants of the demand for routine primary, specialist mental health and emergency care services over time. This prison setting with comprehensive health screening, removal of financial barriers to care and a standardized nurse-led gatekeeping system provides novel insights into the relative importance of health status, socio-demographic characteristics, penal history and the prison environment for a marginalized, high risk population difficult to research. The longitudinal analysis assesses the persistence of prior acute somatic and psychiatric related events on future utilization across the different care specialties.

The findings that female prisoners show higher nursing, mental care and emergency care are consistent with the epidemiological evidence and previous studies underlining the greater need of mental and social support for female prisoners (Jaquier, Neri, Augsburger, & Clair, 2019; Lindquist & Lindquist, 1999; Moschetti et al., 2017; Nowotny, 2016). GP consultations increase with age; however, emergency admissions are higher for the youngest adults (18–25), and mental healthcare was also lower amongst older female inmates. Hence, the deprivation of the prison setting may put younger adults, and in particular females, at higher risk of health complications (Slotboom, Kruttschnitt, Bijleveld, & Menting, 2011). As chronic health conditions are highly correlated with age, disease indicators may partly capture the effect of age-related morbidity in our estimations.

Studies in the community setting of racial, ethnic or cultural differences in HCU suffer from the difficulty of isolating the influence of cultural differences on HCU from problems with diagnosing, accessing and financing care. The prison context of the canton of Vaud minimizes these influences along with other socio-economic factors that impact HCU (Mayberry, Mili, & Ofili, 2000). After controlling for health needs, results show significant differences of healthcare utilization by the prisoner’s country of origin. Inmates from Africa, Eastern Europe and the Americas use less nursing, mental health and emergency services per stay. Detainees with Swiss as well as North African origins require more mental healthcare. Individuals with prior health insurance status, which is a proxy for higher socio-economic status also demand more mental healthcare, which may indicate difficulties adjusting to prison. Racial disparities in the use of health services are also found in the US prisons (Nowotny, 2016, 2017). A history of discrimination by the criminal justice and healthcare systems can results in a loss of trust by minorities and may explain a reluctance to utilize healthcare services (Whetten et al., 2006). Our findings suggest that prison life differentially affects the health of certain ethnic groups who show a higher threshold for using healthcare, and capacity to support the prison environment. Differences in the reporting of pain, ailments or impairments as well as expectations about the benefits of health services (Krupic et al., 2019) may contribute to explain this result. Disparities in health literacy, communication problems due to languages or fear of stigma, a lack of trust in the system and concerns about medical confidentiality could be attributable to these differences. Swiss federal medical guidelines state that specific ethno-socio-cultural aspects should be addressed by medical teams in order to promote equity in access and in HCU among prisoners (Swiss Academy of Medical Sciences, 2015), hence our findings indicate greater attention should be focused on ensuring the care is adequate of some ethnic groups.

Health status related variables are found to be good predictors of HCU. Coefficients for chronic somatic and mental chronic diseases exhibit expected signs and magnitudes observed in previous studies (Moschetti et al., 2017). Inmates with schizophrenia, personality, or neurotic disorders are strongly associated with a higher (between 1.5 to 2 times greater) utilization of emergency services, a finding in line with the literature in the community setting showing higher use of emergency department by individuals suffering from these disorders, especially during a crisis (Digel Vandyk, Young, MacPhee, & Gillis, 2018). These illnesses often require mental follow-up, which, if insufficient, may lead to severe complications and the need for emergency intervention. There was also high demand for primary care services for prisoners diagnosed with psychiatric illnesses. Drug-related offenders consume less health services. Both drug sellers and users are included in this category, but as we control for drug abuse problems this likely reflects healthcare needs of drug dealers. This could reflect differences in preferences for health and related need for healthcare services. Drug sellers may be less risk averse and have a preference for the immediate future, hence health concerns may be less of a priority. They could find it easier to adjust to the prison environment and possibly find favorable positions in the social hierarchy. Prior to imprisonment, they may also have been relatively more active, healthy and wealthy.

Nurses are the most frequently consulted health professionals with 5 and 3 times more as many visits than GPs and mental health professionals respectively, which is consistent with their gatekeeper role in redirecting prisoners to relevant medical professionals and advanced practice role in supporting prisoners with routine care and prescribing from a controlled list of medications. Recent studies from the community show that access to advanced nurse practitioners can reduce emergency department visits (Alexander, Currie, & Schnell, 2019). We find that more nurse visits in the previous period is not associated with a reduction in emergency utilization, but more GP visits, which implies access to primary care is not problematic in avoiding emergency consultations. Complementarity between GP consultations and mental care utilization may be explained by the fact that GPs in Vaud prisons do not prescribe psychotropic drugs and patients need a psychiatric visit to approve medication. There is evidence of substitution between psychiatric care and the utilization of GPs and nurses, implying psychiatric support might reduce somatic health risks or severity of illness. Our study confirms the importance of past utilization in predicting future use of outpatient healthcare, which has been established by other studies, although they tend to analyze healthcare costs, and use of inpatient care (Clewley, Rhon, Flynn, Koppenhaver, & Cook, 2018; Cucciare & O'Donohue, 2006; Dusheiko, Gravelle, Martin, Rice, & Smith, 2011; Ellis & McGuire, 2007).

Amongst prisoners with multiple stays, we observe a persistence of HCU associated with previous acute somatic illnesses and risky or troubling behaviors, especially acting out events. Despite subsequent increases in the use of nurse and mental healthcare services following these events, the use of emergency services remains elevated (+ 17%). There is also persistence of past emergency HCU, which increases emergency use by 5% in the following period as well as primary care utilization, indicating the need for routine follow-up.

Total time spent in prison reduces all types of HCU with each additional year in prison associated with a 6.4% to 13.4% decrease in HCU depending on the specialty. Conversely, each new detention in prison increases HCU, in particular emergency visits, while remaining in pretrial detention is associated with notably higher mental health use. Many prisoners present with multiple health conditions that have been neglected, and prison provides the first access to healthcare for a long time. Hence, the observed reduction in HCU over time may reflect improved health status and lower care needs after initial treatments. Prisoners, especially those with long stays, will have repeated contacts with the same health care professionals, which can improve the (agency) relationship between physicians and patients and treatment compliance (Gafni, Charles, & Whelan, 1998; Rochaix, 1998; Stewart, 1995). When prisoners are released most are not cured and require continuity of care with outside services. However, our results suggest there is inadequate community-based follow-up as recidivists’ health status is likely to have worsened (Young et al., 2015), so special attention to those coming back in prison is required, especially if they suffer from severe mental problems (Jarrett et al., 2012; National Audit Office, 2017). During pretrial detention, and exacerbated upon prison entry, prisoners are exposed to anxiety, interpersonal conflict and sleeping problems due to overcrowding and long periods of confinement (they can spend more than 22 h a day in their cell) (Baćak, Andersen, & Schnittker, 2018). When convicted, inmates have the opportunity to be more active, partake in education programs to improve health literary and to work. This may contribute to improved health and well-being and substitute for healthcare (Gage & Goldfrank, 1985). A Norwegian study found contrasting findings with inmates in remand or pretrial detention having less contact with healthcare professionals than those serving sentences (Nesset, Rustad, Kjelsberg, Almvik, & Bjorngaard, 2011).

Three out of the four prisons in the canton of Vaud were operating at over the official capacity between 2013 and 2017 (mean capacity rates overall in Switzerland are 94%) and inmate turnover increased in most of facilities. Greater prisoner density would favor propagation of infectious and parasitic diseases (Massoglia & Remster, 2019), consistent with our finding of higher demand for GP and nurse consultations. The loss of privacy and increase in stress from overcrowding could also worsen mental health and induce violent and self-harm behaviors (Baggio et al., 2018; Preti & Cascio, 2006). However, our results find overcrowding associated with lower demand for mental and emergency HCU. Overpopulation implies greater competition for existing services, which could lead to unmet mental care needs due to insufficient resources, and higher thresholds for requiring emergency care. A greater inflow of prisoners during the year, however, is associated with higher demand for most services. Given that new prisoners are more demanding, this may reflect time pressures on existing resources created by sudden fluctuations in demand. The M1 model with prison fixed effects finds an increase in expenditure on health services staff increases utilization of mental health services, implying resource constraints may reduce access. Utilization of all health care services was higher in Bois-Mermet which is an old prison with high over-capacity and houses pretrial and transit prisoners with little time out of cells. Conversely, in Plaine de l’Orbe housing convicted prisoners with opportunity to work and exercise, utilization of psychiatric care is in particular, lower. In the two levels models (M2) GP and nurse HCU was higher, and emergency admissions significantly lower. There was also a negative association between expenditure on staff and mental healthcare, which may be an indication that better resourced prisons have better prisoner well-being. The estimations suggest that a CHF 500′000 increase in expenditure on staff salary would reduce emergency interventions by 31% per stay of prisoner. Provided the high organizational, human and financial costs associated with emergency interventions in prison, this would suggest that investment in additional onsite personal resources might improve cost-effectiveness of prison healthcare systems. Further research is needed on this topic.

Our study has some limitations. The external validity of the findings is a concern. The setting of the Canton of Vaud is unique**,** and results may not be fully representative at the national or international level. However, the prison setting is arguable more uniform than the community, removing some socio-economic and environmental barriers to accessing healthcare. Despite the large sample of prisoner observations our prison level variables are measured across only 4 prisons for few years, and lack of variability in prison conditions may reduce the precision of the estimates. For that reason and data availability we did not assess the impact of other prison level variables such as prison guard levels, age of the facility, other health and education initiatives. Instead, these unmeasured differences are taken into account through prison fixed or random effects. The study does not make strict causal claims regarding the effects of the factors analyzed, however, it provides valuable insights into the HCU patterns of a marginalized population whose healthcare utilization is understudied. The data does not explicitly capture the chronology of diagnoses and each healthcare services utilization from the start of each prison stay, which would allow for an analysis of the evolution of the health status and utilization from onset of disease diagnosis. While this analysis focuses on the three important types of healthcare covering primary, mental and emergency, it does not include other specialist outpatient care or inpatient hospital stays all provided outside the prison walls. Further research is required on referrals to specialist care and planned hospitalizations. However, the study covers 92% of the total volume of healthcare services provided onsite, allowing us to infer important patterns of HCU by prisoners.

## Conclusion

The prison setting provides an opportunity to diagnose and treat significant unmet health needs, and to prioritize care through the gatekeeping system. Investing to improve access and quality of care to ensure prisoners at higher risk receive effective treatments during incarceration and continuity of care inside and outside of prison may have wider public health benefits. Young, and in particular, female prisoners have greater mental health and emergency care use, while most ethnic minorities under-utilize care. Hence, prison services and the criminal justice system should consider how confinement may exacerbate health care needs and how to ensure adequate care. Adverse health events lead to persistent HCU over time and across different services, requiring effective co-ordination of health services as well as interventions that reduce the need for acute care. A broader perspective that considers the importance of the prison environment and penal circumstances on the healthcare needs for a medically complex and socially vulnerable population is required. Pretrial detention in particular creates a significant burden on health services as well as adding to over-crowding. Our findings that repeat offenders require more health care than long-staying prisoners points to the benefits of comprehensive screening, health needs assessment and access to care on prison entry. A high prisoner turnover increases HCU and the demands on prison health services. The relationship between the criminal justice system and the prison healthcare system should be more integrated to improve the planning of service delivery and inform prison policy.

## Supplementary Information

**Additional file 1: Appendix A1.** Results of the multilevel GLM regression models (M2) of prisoners’ generalist (GP), nursing, mental and emergency healthcare utilization. **Appendix A2.** Results of the dynamic regression models (M3) of prisoners’ generalist (GP), nursing, mental and emergency healthcare utilization.

## Data Availability

Regarding data availability, ethical and legal restrictions prohibit the authors from making the data publicly available. Indeed, these data contain very sensitive judiciary information and medical information on individual prisoners. However, interested researchers who wish to request access to the data may contact M. Didier Delessert, head of the Service of Correctional Medicine and Psychiatry (SMPP), University Hospital of Lausanne (CHUV), Lausanne, Switzerland. The data can be made available upon request provided that the necessary ethical and legal obligations that apply to the data are fulfilled.

## Abbreviations

CHUV: University Hospital of Lausanne (Centre Hospitalier Universitaire Vaudois)
EPO: Etablissements de la Plaine de l’Orbe
GP: General Practitioner
ICD-10: International Statistical Classification of Diseases and Related Health Problems Version 10
SCC: Swiss Criminal Code
SMPP: Service of Psychiatry and Correctional Medicine (Service de Médecine et Psychiatrie Pénitentiaire)
