# Supplementary material for: Healthcare in a pure gatekeeping system: utilization of primary, mental and emergency care in the prison population over time
Source: Health Justice. 2021 May 13;9:11. doi: 10.1186/s40352-021-00136-8 (PMC8120814; doi:10.1186/s40352-021-00136-8)
Supplement: Supplementary file 1 — Additional file 1: Appendix A1. Results of the multilevel GLM regression models (M2) of prisoners’ generalist (GP), nursing, mental and emergency healthcare utilization. Appendix A2. Results of the dynamic regression models (M3) of prisoners’ generalist (GP), nursing, mental and emergency healthcare utilization. [file 40352_2021_136_MOESM1_ESM.docx]

**Appendix A1**

| **Results of the multilevel GLM regression models (M2) of prisoners’ generalist (GP), nursing, mental and emergency healthcare utilization** | | | | | | | | |
| --- | --- | --- | --- | --- | --- | --- | --- | --- |
|  | **GP** | | **Nurse** | | **Mental** | | **Emergency** | |
|  | **IRR** | **SE** | **IRR** | **SE** | **IRR** | **SE** | **IRR** | **SE** |
|  |  |  |  |  |  |  |  |  |
| Female | 1.281* | 0.180 | 1.376*** | 0.120 | 2.313*** | 0.362 | 2.893*** | 0.874 |
| Married | 1.045 | 0.035 | 0.954** | 0.022 | 0.945 | 0.047 | 1.119 | 0.121 |
| Health insurance | 1.013 | 0.034 | 0.969* | 0.017 | 1.282*** | 0.055 | 0.958 | 0.095 |
| **Age** |  |  |  |  |  |  |  |  |
| 25 to 39 | 1.047 | 0.037 | 0.968 | 0.020 | 1.050 | 0.052 | 0.801** | 0.086 |
| 40 to 49 | 1.049 | 0.049 | 0.960 | 0.028 | 0.936 | 0.062 | 0.677*** | 0.102 |
| 50 and older | 1.118* | 0.068 | 0.959 | 0.037 | 0.915 | 0.078 | 0.967 | 0.180 |
| Female 25 to 39 | 1.008 | 0.150 | 0.877 | 0.076 | 0.837 | 0.142 | 0.558* | 0.187 |
| Female 40 to 49 | 1.151 | 0.196 | 1.107 | 0.124 | 0.641** | 0.130 | 0.610 | 0.245 |
| Female 50 and older | 1.370* | 0.258 | 0.891 | 0.116 | 0.709 | 0.166 | 0.418* | 0.194 |
| **Origin (+)** |  |  |  |  |  |  |  |  |
| Switzerland | 0.944 | 0.047 | 1.116*** | 0.040 | 1.251*** | 0.080 | 0.863 | 0.122 |
| Africa | 0.937 | 0.045 | 0.831*** | 0.029 | 0.466*** | 0.032 | 0.463*** | 0.071 |
| North Africa | 1.089* | 0.055 | 1.082** | 0.040 | 1.105 | 0.075 | 1.269* | 0.180 |
| Middle East | 1.132 | 0.100 | 1.188** | 0.081 | 1.239* | 0.144 | 1.254 | 0.305 |
| Eastern Europe | 0.979 | 0.045 | 0.811*** | 0.028 | 0.631*** | 0.041 | 0.729** | 0.102 |
| America | 0.918 | 0.082 | 1.059 | 0.068 | 0.915 | 0.110 | 0.549** | 0.161 |
| Asia | 0.740** | 0.102 | 0.934 | 0.094 | 1.009 | 0.179 | 0.721 | 0.297 |
| **Chronic somatic conditions** |  |  |  |  |  |  |  |  |
| Infectious | 1.573*** | 0.078 | 1.082*** | 0.027 | 1.061 | 0.072 | 1.294 | 0.206 |
| Skin | 1.358*** | 0.054 | 1.003 | 0.017 | 0.851*** | 0.047 | 0.937 | 0.124 |
| Musculoskeletal | 1.426*** | 0.055 | 0.978 | 0.016 | 0.975 | 0.051 | 1.044 | 0.130 |
| Digestive | 1.416*** | 0.066 | 1.093*** | 0.020 | 0.972 | 0.062 | 1.306* | 0.193 |
| Circulatory | 1.446*** | 0.076 | 1.273*** | 0.028 | 0.927 | 0.068 | 2.431*** | 0.386 |
| Endocrine | 1.349*** | 0.070 | 1.277*** | 0.027 | 1.044 | 0.073 | 1.152 | 0.197 |
| Respiratory | 1.257*** | 0.063 | 0.947*** | 0.020 | 0.959 | 0.064 | 1.031 | 0.165 |
| Nervous | 1.358*** | 0.105 | 1.158*** | 0.036 | 1.066 | 0.110 | 1.240 | 0.283 |
| **Chronic addictions** |  |  |  |  |  |  |  |  |
| Alcohol | 0.949 | 0.045 | 1.026 | 0.021 | 1.702*** | 0.096 | 0.673*** | 0.101 |
| Drugs | 1.078* | 0.044 | 1.044*** | 0.017 | 2.776*** | 0.138 | 1.438*** | 0.174 |
| Pharmaceuticals | 0.949 | 0.061 | 0.815*** | 0.022 | 1.403*** | 0.106 | 0.879 | 0.164 |
| **Chronic mental conditions** |  |  |  |  |  |  |  |  |
| Schizophrenia | 0.908 | 0.057 | 1.143*** | 0.031 | 6.088*** | 0.447 | 1.504** | 0.272 |
| Mood | 1.296*** | 0.099 | 1.135*** | 0.036 | 3.097*** | 0.287 | 1.772*** | 0.379 |
| Neurotic | 1.092** | 0.043 | 1.076*** | 0.018 | 3.324*** | 0.166 | 1.580*** | 0.186 |
| Behavioral | 1.039 | 0.097 | 0.938 | 0.037 | 2.543*** | 0.296 | 0.412** | 0.169 |
| Personality | 1.084** | 0.043 | 1.040** | 0.016 | 2.624*** | 0.126 | 2.230*** | 0.262 |
| Retardation | 0.959 | 0.074 | 1.075** | 0.032 | 1.481*** | 0.137 | 1.381 | 0.277 |
| Chronic comorbidities | 1.074*** | 0.023 | 1.042*** | 0.008 | 1.057** | 0.028 | 1.037 | 0.069 |
| **Year dummies** |  |  |  |  |  |  |  |  |
| 2014 | 0.842*** | 0.038 | 0.932*** | 0.014 | 0.906** | 0.045 | 0.621*** | 0.071 |
| 2015 | 0.832*** | 0.037 | 0.760*** | 0.014 | 1.130** | 0.062 | 0.501*** | 0.061 |
| 2016 | 0.927* | 0.040 | 0.677*** | 0.014 | 1.212*** | 0.067 | 0.358*** | 0.046 |
| 2017 | 1.018 | 0.055 | 0.645*** | 0.015 | 1.354*** | 0.086 | 0.435*** | 0.062 |
| **Prisoner characteristics** |  |  |  |  |  |  |  |  |
| Preventive detention | 1.041 | 0.033 | 1.177*** | 0.017 | 1.404*** | 0.057 | 1.104 | 0.105 |
| Mandatory psych. treat. | 0.936 | 0.063 | 0.988 | 0.026 | 1.429*** | 0.112 | 1.257 | 0.234 |
| Time in prison | 0.958*** | 0.009 | 0.873*** | 0.006 | 0.933*** | 0.012 | 0.881*** | 0.025 |
| Number of stays | 1.055*** | 0.017 | 1.061*** | 0.008 | 1.006 | 0.021 | 1.328*** | 0.056 |
| **Type of offense (++)** |  |  |  |  |  |  |  |  |
| Violent | 0.838*** | 0.049 | 0.954 | 0.037 | 1.195** | 0.092 | 0.924 | 0.155 |
| Sexual | 0.991 | 0.050 | 1.039 | 0.034 | 1.615*** | 0.106 | 1.007 | 0.150 |
| Drug related | 0.950* | 0.027 | 0.918*** | 0.015 | 0.862*** | 0.033 | 0.883 | 0.077 |
| **Prison characteristics** |  |  |  |  |  |  |  |  |
| Occupancy | 1.334** | 0.157 | 1.282*** | 0.080 | 0.416*** | 0.047 | 0.558** | 0.136 |
| Prison entries in the year | 1.000 | 0.000 | 1.001*** | 0.000 | 1.000** | 0.000 | 1.000 | 0.000 |
| Full-time equivalent cost | 1.128 | 0.111 | 1.092 | 0.061 | 0.670*** | 0.048 | 0.374*** | 0.061 |
|  |  |  |  |  |  |  |  |  |
| Constant | 0.006*** | 0.001 | 0.042*** | 0.006 | 0.022*** | 0.005 | 0.007*** | 0.004 |
|  |  |  |  |  |  |  |  |  |
| Observations | 10,136 |  | 10,136 |  | 10,136 |  | 10,136 |  |
| Number of individuals |  |  |  |  |  |  |  |  |
| Distribution | Negbin |  | Poisson |  | Negbin |  | Negbin |  |
| Pseudo R2 | 0.316 |  | 0.235 |  | 0.107 |  | 0.081 |  |
| Incidence rate ratios (IRR) and standard errors (SE) reported,*** p<0.01, ** p<0.05, * p<0.1. Reference categories are (+) Western Europe, (++) Other offenses. | | | | | | | | |
|  |  |  |  |  |  |  |  |  |

**Appendix A2**

| **Results of the dynamic regression models (M3) of prisoners’ generalist (GP), nursing, mental and emergency healthcare utilization** | | | | | | | | |
| --- | --- | --- | --- | --- | --- | --- | --- | --- |
|  | **GP** | | **Nurse** | | **Mental** | | **Emergency** | |
|  | **IRR** | **SE** | **IRR** | **SE** | **IRR** | **SE** | **IRR** | **SE** |
|  |  |  |  |  |  |  |  |  |
| Female | 0.998 | 0.192 | 0.857 | 0.135 | 1.851*** | 0.367 | 1.276 | 0.482 |
| Married | 1.035 | 0.042 | 0.926** | 0.029 | 0.946 | 0.053 | 0.969 | 0.134 |
| Health insurance | 0.986 | 0.038 | 1.005 | 0.028 | 1.132*** | 0.049 | 0.931 | 0.104 |
| **Age** |  |  |  |  |  |  |  |  |
| 25 to 39 | 1.019 | 0.045 | 0.990 | 0.032 | 0.948 | 0.052 | 0.851 | 0.108 |
| 40 to 49 | 1.028 | 0.059 | 0.962 | 0.042 | 0.919 | 0.067 | 0.776 | 0.140 |
| 50 and older | 1.063 | 0.077 | 0.962 | 0.054 | 0.967 | 0.086 | 1.135 | 0.250 |
| Female 25 to 39 | 1.270 | 0.261 | 1.193 | 0.200 | 0.780 | 0.165 | 0.707 | 0.300 |
| Female 40 to 49 | 1.543* | 0.346 | 1.470** | 0.272 | 0.749 | 0.176 | 1.095 | 0.537 |
| Female 50 and older | 1.667** | 0.403 | 1.383 | 0.285 | 0.494*** | 0.135 | 0.564 | 0.307 |
| **Origin (+)** |  |  |  |  |  |  |  |  |
| Switzerland | 0.930 | 0.054 | 1.032 | 0.047 | 1.267*** | 0.084 | 1.122 | 0.192 |
| Africa | 0.939 | 0.053 | 0.899** | 0.040 | 0.591*** | 0.045 | 0.726* | 0.141 |
| North Africa | 1.094 | 0.065 | 1.118** | 0.052 | 1.151** | 0.082 | 1.502** | 0.252 |
| Middle East | 0.989 | 0.101 | 1.077 | 0.087 | 1.160 | 0.134 | 1.215 | 0.345 |
| Eastern Europe | 1.015 | 0.057 | 0.967 | 0.043 | 0.744*** | 0.056 | 0.908 | 0.166 |
| America | 0.961 | 0.100 | 1.042 | 0.086 | 1.040 | 0.134 | 0.798 | 0.292 |
| Asia | 0.783* | 0.116 | 0.971 | 0.112 | 0.947 | 0.166 | 1.483 | 0.605 |
| **Chronic somatic conditions** |  |  |  |  |  |  |  |  |
| Infectious | 1.529*** | 0.091 | 1.115** | 0.052 | 1.045 | 0.076 | 1.423* | 0.270 |
| Skin | 1.376*** | 0.059 | 1.068** | 0.036 | 0.881** | 0.047 | 0.886 | 0.133 |
| Musculoskeletal | 1.416*** | 0.058 | 1.096*** | 0.035 | 1.063 | 0.049 | 0.939 | 0.127 |
| Digestive | 1.354*** | 0.064 | 1.098*** | 0.040 | 0.984 | 0.054 | 1.259 | 0.188 |
| Circulatory | 1.195*** | 0.068 | 1.380*** | 0.060 | 0.893 | 0.063 | 1.714*** | 0.310 |
| Endocrine | 1.309*** | 0.072 | 1.216*** | 0.051 | 1.004 | 0.066 | 1.330 | 0.239 |
| Respiratory | 1.276*** | 0.068 | 1.063 | 0.043 | 0.981 | 0.061 | 0.962 | 0.168 |
| Nervous | 1.278*** | 0.106 | 1.281*** | 0.074 | 0.852 | 0.084 | 1.072 | 0.271 |
| **Chronic addictions** |  |  |  |  |  |  |  |  |
| Alcohol | 0.976 | 0.053 | 0.998 | 0.039 | 1.274*** | 0.070 | 0.847 | 0.142 |
| Drugs | 1.070 | 0.049 | 1.126*** | 0.038 | 1.957*** | 0.097 | 1.352** | 0.183 |
| Pharmaceuticals | 0.945 | 0.065 | 0.921* | 0.044 | 1.214*** | 0.079 | 0.872 | 0.167 |
| **Chronic mental conditions** |  |  |  |  |  |  |  |  |
| Schizophrenia | 0.916 | 0.065 | 1.215*** | 0.061 | 3.152*** | 0.209 | 1.149 | 0.242 |
| Mood | 1.098 | 0.091 | 1.038 | 0.064 | 1.929*** | 0.157 | 1.072 | 0.252 |
| Neurotic | 1.127*** | 0.052 | 1.098*** | 0.038 | 1.813*** | 0.093 | 1.419** | 0.196 |
| Behavioral | 1.064 | 0.124 | 0.999 | 0.083 | 1.461*** | 0.187 | 0.386* | 0.206 |
| Personality | 1.051 | 0.047 | 1.081** | 0.035 | 2.039*** | 0.098 | 2.146*** | 0.276 |
| Retardation | 1.057 | 0.079 | 1.099* | 0.059 | 1.191** | 0.090 | 1.490** | 0.272 |
|  |  |  |  |  |  |  |  |  |
| Chronic comorbidities | 1.011 | 0.024 | 0.998 | 0.019 | 1.019 | 0.025 | 0.945 | 0.071 |
| **Year dummies** |  |  |  |  |  |  |  |  |
| 2014 | 0.775*** | 0.058 | 0.764*** | 0.037 | 0.844** | 0.066 | 0.857 | 0.161 |
| 2015 | 0.776*** | 0.060 | 0.700*** | 0.035 | 0.925 | 0.077 | 0.592*** | 0.113 |
| 2016 | 0.805*** | 0.061 | 0.635*** | 0.032 | 0.920 | 0.073 | 0.359*** | 0.069 |
| 2017 | 0.848* | 0.073 | 0.581*** | 0.034 | 1.007 | 0.095 | 0.503*** | 0.116 |
| **Prisoner characteristics** |  |  |  |  |  |  |  |  |
| Preventive detention | 1.010 | 0.049 | 1.161*** | 0.038 | 1.381*** | 0.074 | 1.019 | 0.142 |
| Mandatory psych. treat. | 0.870** | 0.059 | 0.959 | 0.047 | 1.054 | 0.068 | 1.140 | 0.207 |
| Time in prison | 0.929*** | 0.010 | 0.912*** | 0.008 | 0.935*** | 0.012 | 0.879*** | 0.030 |
| Number of stays | 1.064*** | 0.017 | 1.103*** | 0.013 | 1.061*** | 0.020 | 1.332*** | 0.054 |
| **Type of offense (++)** |  |  |  |  |  |  |  |  |
| Violent | 0.886** | 0.051 | 0.913** | 0.042 | 1.139** | 0.075 | 0.835 | 0.144 |
| Sexual | 0.987 | 0.052 | 1.021 | 0.043 | 1.356*** | 0.080 | 0.827 | 0.131 |
| Drug related | 1.021 | 0.034 | 1.011 | 0.025 | 0.939 | 0.038 | 0.841* | 0.083 |
| **Prison dummies (+++)** |  |  |  |  |  |  |  |  |
| Bois-Mermet | 1.278 | 0.242 | 1.170 | 0.143 | 1.646** | 0.345 | 4.260*** | 2.304 |
| La Tuilière | 1.087 | 0.208 | 0.712** | 0.094 | 1.066 | 0.228 | 4.932*** | 2.867 |
| Plaine de l'Orbe | 1.342 | 0.438 | 0.735 | 0.160 | 0.588 | 0.208 | 1.833 | 1.786 |
| **Prison characteristics** |  |  |  |  |  |  |  |  |
| Occupancy | 1.192 | 0.305 | 0.710** | 0.118 | 0.336*** | 0.085 | 0.626 | 0.420 |
| Prison entries in the year | 1.000 | 0.000 | 1.000 | 0.000 | 1.000 | 0.000 | 1.003*** | 0.001 |
| Full-time equivalent cost | 0.993 | 0.208 | 1.055 | 0.145 | 1.245 | 0.292 | 0.993 | 0.630 |
| **Acute events and HCU Lagged** |  |  |  |  |  |  |  |  |
| Crisis events | 0.985* | 0.009 | 0.978*** | 0.006 | 0.990 | 0.009 | 0.941*** | 0.020 |
| Acting out events | 1.002 | 0.020 | 1.036*** | 0.013 | 1.051*** | 0.018 | 1.170*** | 0.043 |
| Acute somatic conditions | 1.077*** | 0.022 | 1.041*** | 0.015 | 0.979 | 0.023 | 0.976 | 0.063 |
| GP consultations | 1.014*** | 0.004 | 1.016*** | 0.003 | 1.012** | 0.005 | 1.017 | 0.012 |
| Nurse consultations | 1.002*** | 0.001 | 1.000 | 0.001 | 0.999 | 0.001 | 1.003 | 0.003 |
| Mental consultations | 0.995** | 0.002 | 0.996** | 0.002 | 1.020*** | 0.002 | 1.004 | 0.006 |
| Emergency consultations | 1.035** | 0.016 | 1.019* | 0.010 | 0.984 | 0.016 | 1.054* | 0.032 |
| Length of stay | 1.000** | 0.000 | 0.999*** | 0.000 | 0.999*** | 0.000 | 0.999** | 0.001 |
| Baseline visits | 1.008*** | 0.001 | 1.011*** | 0.001 | 1.006*** | 0.001 | 1.012*** | 0.002 |
|  |  |  |  |  |  |  |  |  |
| Constant | 0.008*** | 0.003 | 0.042*** | 0.011 | 0.009*** | 0.004 | 0.001*** | 0.001 |
|  |  |  |  |  |  |  |  |  |
| Observations | 5,208 |  | 5,208 |  | 5,208 |  | 5,208 |  |
| Number of individuals | 2,594 |  | 2,594 |  | 2,594 |  | 2,594 |  |
| Distribution |  |  |  |  |  |  |  |  |
| Pseudo R2 | 0.364 |  | 0.411 |  | 0.401 |  | 0.216 |  |
| Incidence rate ratios (IRR) and standard errors (SE) reported,*** p<0.01, ** p<0.05, * p<0.1. Reference categories are (+) Western Europe, (++) Other offenses, (+++) La Croisée. | | | | | | | | |
|  |  |  |  |  |  |  |  |  |
